# Plasma brain-derived neurotrophic factor is higher after combat training (Randori) than incremental ramp test in elite judo athletes

**DOI:** 10.1590/1414-431X20198154

**Published:** 2019-04-08

**Authors:** B. Schor, S. Gomes da Silva, A.A. de Almeida, C.A.B. Pereira, R.M. Arida

**Affiliations:** 1 Departamento de Ortopedia, Instituto Vita, São Paulo, SP, Brasil; 2 Hospital Israelita Albert Einstein, São Paulo, SP, Brasil; 3 Departamento de Fisiologia, Universidade Federal de São Paulo, São Paulo, SP, Brasil; 4 Departamento de Estatística, Universidade de São Paulo, São Paulo, SP, Brasil; 5 Instituto Federal Goiano, Campus Ceres, Ceres, GO, Brasil

**Keywords:** Judo, BDNF, Elite athletes, Neuroplasticity, Randori, Exercise

## Abstract

Elite judo demands high levels of physical and psychological skills. The brain-derived neurotrophic factor (BDNF) may be of particular interest in sports medicine for its ability to promote neuroplasticity. We investigated the plasma BDNF before and after a judo training session (Randori) and the maximal incremental ramp test (MIRT) in athletes from the Brazilian national judo team and compared the results between both exercise stimuli and sexes. Fifty-six elite judo athletes were recruited who performed each task on a separated day. Anthropometric, physiological, athletic parameters, and plasma BDNF levels were measured before and after the tasks (Randori and MIRT). The groups presented similar anthropometric and physiological characteristics at baseline for the two tasks. All athletes reached maximal performance for the tasks. Plasma levels of BDNF increased significantly after Randori and MIRT for all subjects, both men and women. When comparing both exercise stimuli, a greater increase in BDNF was observed after Randori. There was no significant difference in the delta BDNF between sexes. Our findings indicate that training specificity of sport gestures influenced the increase of blood BDNF levels.

## Introduction

Physical exercise has long been recognized to have a positive impact on brain structure and cognitive function in humans (1). A number of molecular systems participate in the beneficial effects of exercise on the brain (1,2). Neurotrophic factors play a key role in the effect of exercise by regulating neuroplasticity (2). The brain-derived neurotrophic factor (BDNF) plays a key role in the brain during development and adult life (3–9), retarding neuronal cell death, inducing regeneration, and stimulating neuron survival in the brain (10,11).

Human studies have shown that serum BDNF levels are elevated in response to aerobic exercise (12,13), and exercise intensity is correlated with BDNF levels in healthy individuals (14,15). While Yarrow et al. (16) found increased BDNF after resistance training, Correia et al. (17) reported no changes in BDNF levels after a strength exercise session. Increased BDNF levels have also been associated with physical activity and cognition. In animal studies, increased BDNF brain expression, especially in the hippocampus, has been observed following different types of physical activity (18–20), showing a strong relationship with memory performance (21), thus suggesting that BDNF may be a potential candidate to mediate the long-term effects of exercise in the brain. Since BDNF is affected by physical training (12,13), increased BDNF may depend on the level of physical effort and individual fitness, which might be attributed to an individual's training status. In this regard, few studies have evaluated BDNF levels in athletes. In a study conducted by Zoladz et al. (12), the basal BDNF from athletes competing in various athletic events (sprinters, jumpers, and distance runners) was significantly higher than untrained subjects. In another investigation, basal blood BDNF was significantly elevated in international and national runners compared to sedentary individuals (22). However, it is important to point out that the majority of studies have reported altered BDNF following simple (cyclic) sports gestures (23–25). Few investigations have analyzed the impact of complex sports gestures or multimodal activity on neurotrophins (for review, see 26). Jacini et al. (27) has demonstrated an association between the practice of a physical exercise involving complex motor planning and control and higher grey matter tissue density in brain regions responsible for these tasks.

Elite judo demands high levels of physical and psychological skills and carries a high risk of injury. It is a complex sport gesture that demands a multimodal training schedule with high cognitive function demand. Thus, it is important to identify molecules and develop new methods and techniques to prevent injuries, as well as specify biological markers to evaluate performance, training load, and training efficacy to increase the performance of elite judo athletes (28). Studies conducted with high-performance athletes are unusual because of the difficulty of avoiding interference with their training and coordinating with the sports calendar. Therefore, investigations in elite athletes can supply additional information regarding athletes' training status or their performance level.

This study was designed to assess, for the first time, the effect of complex sports gestures (acyclic) in a specific sport modality and simple sport gestures (cyclic) on the BDNF blood levels in elite athletes and with respect to sex. For this purpose, a judo training session was compared with incremental exercise to the point of exhaustion (ramp test) on the BDNF blood levels in a group of Brazilian elite judo athletes.

## Material and Methods

### Human volunteers

Fifty-six elite judo athletes were recruited for this study, which was approved by the Ethics Committee of the Universidade Federal de São Paulo, Brazil (#698.019). All volunteers were informed of the aims of the study and received written, detailed explanations about the experiments, the potential types of discomfort, the risks, and the procedures employed in the investigation; they then signed an informed consent to participate in the study.

The inclusion criteria included holding a black belt on the Brazilian judo team, being an international-level competitor (i.e., an athlete participating in the Olympic Games, world championships and International Judo Federation Grand Slams), 10 years or more of judo practice and more than 15 h training per week. The exclusion criteria included athletes with recent injuries and concussions, individuals who were taking medication for psychological or psychiatric treatment, alcoholics, smokers, and those who did not complete all tests or failed to provide all of the blood samples.

**Figure 1:**
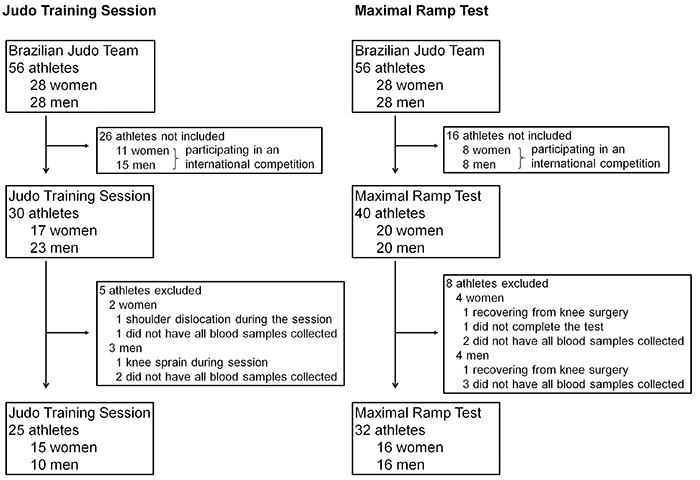
Flow diagram of the subjects included in this study.

The same population was tested for both tasks. The final sample consisted of 25 athletes in the judo training session (Randori) during the first year of the 2016 Olympic cycle (15 women, age 23.3±0.8 years, height 163.3±01.8 cm, weight 63.7±3.8 kg and 10 men, age 25.3±1.0 years, height 181.6±3.2 cm, weight 96.1±9.4 kg) and 32 athletes were selected from the Brazilian judo team to be submitted to a ramp test during the second year of the 2016 Olympic cycle (16 women, age 23.3±0.8 years, height 165.9±01.7 cm, weight 66.4±3.6 kg and 16 men, age 24.2±0.8 years, height 177.1±2.8 cm, weight 87.7±6.8 kg); 11 athletes were in both the first and second samples (Figure 1).

### Judo training session (Randori)

The judo training session included a ten-minute warm-up, which consisted of injury prevention exercises. The warm-up was immediately followed by a sequence of eight judo fights (Randori) that were each 3 min long, alternating with 3 min of rest, to total a one-hour training session (Figure 1). Two Brazilian team coaches accompanied all of the training and encouraged the athletes to maintain the intensity of the fights. After the Randori, one minute of high-intensity judo (uticome) strokes were performed in order to attain a heart rate that was comparable to fights during competitions. The researchers did not interfere in the training so that the physiological response of the athletes was as accurate as possible.

### Maximal incremental ramp test (MIRT)

The MIRT consisted of an initial 3 min of walking at 2 W/kg, followed by incremental exercise to exhaustion with 25 W steps every 30 s. It was ensured that subjects had reached maximal performance by fulfilling the following criteria: reaching of maximal heart rate (HRmax: 220 minus age in years), a rating of perceived exertion (RPE) higher than 17, an increased respiratory exchange ratio (RQ) higher than 1.1, a ratio of respiratory minute volume in mL and oxygen uptake during that same minute higher than 30–35, no further increase of the quotient of oxygen uptake in mL and the pulse rate during the same minute. After exhaustion was reached, an active recovery period of 3 min was performed.

### Blood analysis

Blood samples (12 mL divided into 2 tubes of 6 mL each) were taken by venipuncture and collected in a heparin tube after a resting period of 30 min at room temperature (25°C) and 1 min post-effort. The blood was placed on ice to be centrifuged at 3000 *g* for 15 min at 4°C) and the plasma was extracted from a tube and stored in an Eppendorf freezer tube (Invitrogen, USA) at –80°C for further analysis. The plasma BDNF analysis was performed using the ELISA kit E-max^®^ (Promega, USA) according to manufacturer's recommendations. Samples from the studied groups previously stored at –80°C were centrifuged for 5 min at 1500 *g* at 4°C and the supernatant was transferred to a 96-well plate (Corning Costar, USA), coated with anti-BDNF (1:1000), and then incubated for 2 h at room temperature. After this period, the plate was washed with Tris-buffered saline Tween-20 (TBS-T) (Sigma, USA) and incubated with the following antibodies: anti-human (1:500) (Promega, USA) for 2 h and conjugate anti-IgY HRP (1:200) for 1 hour. After these procedures, color reaction with tetramethyl benzidine was quantified in a plate reader at 450 nm (Quick Elisa, Thermo Fisher Scientific, USA). The values are reported in pg/mL.

### Statistical analysis

The normality of the data distribution was assessed by the Shapiro-Wilk test. The variables with normal distribution were compared using parametric tests (Student’s *t*-test) and those that rejected normality were compared using non-parametric Mann-Whitney test for comparison between sexes. Friedman's ANOVA was used for comparing the BDNF response among different times after Randori and MIRT. The Mann-Whitney test was used to identify differences between times. Data are reported as means±SE. Data were analyzed by Microsoft Excel^®^ for Mac 2011 Version 14.5.4 (USA). P<0.05 indicated statistical significance.

## Results

### Characteristics of volunteers

No significant difference between women and men was observed in terms of age, judo training years (JT), and weekly training hours (WTH) for both Randori and MIRT and participants (Table 1). The comparison between both exercise conditions showed that all anthropometric and judo training profiles were similar according to age (P=0.544), height (P=0.573), weight (P=0.950), BMI (P=0.993), JT (P=0.865), and WTH (P=0.142).

**Table 1:** Characteristics of volunteers.

	Judo training session				Ramp test			
	Females(n=15)	Males(n=10)	Total(n=25)	P value	Females(n=16)	Males(n=16)	Total(n=32)	P value
Age (years)	23.3±0.8	25.3±1.0	24.1±0.6	0.154	23.3±0.8	24.2±0.8	23.7±0.6	0.447
Height (cm)	163.3±1.8	181.6±3.2	170.6±2.4	<0.001*	1665.9±1.7	177.1±2.8	171.6±1.9	0.006*
Weight (kg)	63.7±3.8	96.1±9.4	76.6±5.4	0.001*	66.4±3.6	87.7±6.8	77.1±4.3	0.009*
BMI (kg/m^2^)	23.7±1.0	28.6±1.9	25.6±1.1	0.020*	23.9±1.0	27.4±1.2	25.6±0.8	0.010*
JT (years)	14.8±0.5	15.9±0.6	15.2±0.4	0.215	14.8±0.8	15.0±0.9	14.9±0.6	0.864
WTH (hours)	26.0±0.7	26.5±0.8	26.2±0.5	0.649	25.5±0.5	25.2±0.4	25.3±0.3	0.646

Data are reported as means±SE. BMI: body mass index; JT: judo training years; WTH: weekly training hours. *Significant difference between males and females (Mann-Whitney and Student’s *t*-tests).

### Physiological profiles

During Randori and MIRT, no significant difference between women and men were found with respect to HRmax (P=0.313; P=0.791) and the percentage of HRmax according to age (% HRmax) (P=0.063; P=0.428). The maximal oxygen volume (VO_2_ max) in the ramp test was higher in men than women (P=0.006) (Table 2).

**Table 2:** Physiological profiles of the groups for the judo training session and ramp test.

	Judo training session				Ramp test			
	Female(n=15)	Male(n=10)	Total(n=25)	P value	Female(n=16)	Male(n=16)	Total(n=32)	P value
HRmax (bpm)	168.2±0.6	168.4±10.8	168.3±0.5	0.313	188.4±1.8	188.3±2.2	188.3±1.4	0.791
% HR max	85.0±0.4%	86.5±0.6%	85.6±0.0%	0.063	95.7±0.8%	96.3±1.0%	96.0±0.6%	0.428
VO_2_ max					41.8±1.2	47.5±1.3	44.7±1.0	0.006*

Data are reported as means±SE. HRmax: maximal heart rate reached at maximal effort; bpm: beats per minute; %HRmax: percentage of maximal heart rate; VO_2_ max: maximal oxygen volume reported as (mL·kg^-1^·min^-1^). *Significant difference between males and females (Mann-Whitney and Student’s *t*-tests).

### BDNF - Randori

**Figure 2:**
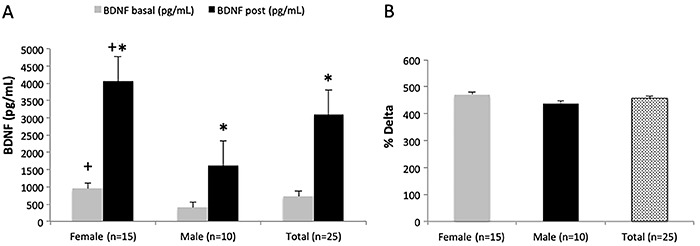
Plasma brain-derived neurotropic factor (BDNF) before (basal) and after the judo training session. **A**, BDNF was higher in females than in males at rest (P=0.001) and after training session (P=0.007). Increase in BDNF levels was observed in the whole sample (P<0.0001) as well as in female (P=0.001) and male groups (P=0.0004). **B**, Delta BDNF was similar between females and males (P=0.801). Data are reported as means±SE. *Statistically significant difference between rest and after training session; ^+^statistically significant difference between males and females (Mann-Whitney and Student’s *t*-tests).

The BDNF levels increased significantly after Randori for the total sample (P<0.0001), for women (P=0.001) and for men (P<0.0004). Female athletes presented higher plasma BDNF at rest (P=0.001) and after Randori compared to male athletes (P=0.007) (Figure 2A). The delta BDNF did not differ significantly between women and men (P=0.801) (Figure 2B).

### BDNF - MIRT

**Figure 3:**
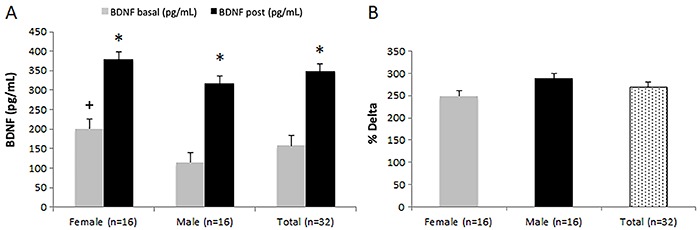
Plasma brain-derived neurotropic factor (BDNF) before (basal) and after maximal incremental ramp test. **A**, Basal BDNF was higher in females than in males (P=0.014). Increase in BDNF levels was observed in the whole sample (P<0.0001) as well as in females (P=0.0004) and males (P<0.0001). **B**, No significant difference was found in delta BDNF between females and males (P=0.393). Data are reported as means±SE. *Statistically significant difference between rest and after training session; ^+^statistically significant difference between males and females (Mann-Whitney and Student’s *t*-tests).

The BDNF levels increased significantly after MIRT for the total sample (P<0.0001), women (P=0.0004), and men (P<0.0001). Female athletes presented higher basal plasma BDNF compared to male athletes (P=0.014). However, no significant difference in BDNF levels between women and men was observed after MIRT (P=0.109) (Figure 3A). The analysis of delta BDNF showed no significant difference between women and men (P=0.393) (Figure 3B).

### Comparison between Randori and MIRT

The athletes who were submitted to MIRT achieved a higher heart rate than those submitted to Randori (P<0.0001). However, all athletes reached 85% of maximal age-predicted heart rate (Table 2). The delta BDNF was higher in the Randori (457.7±24.8%) than the ramp test (268.8±24.8%) (P=0.003) participants.

## Discussion

This study addressed the influence of Randori and incremental exercise to exhaustion on BDNF plasma levels among elite judo athletes. The main and novel findings of this study were that BDNF plasma levels were higher in athletes submitted to Randori than to MIRT. Both types of physical effort induced a significant increase in plasma BDNF. Higher basal BDNF was observed in women compared to men and resulted in a similar increase post-effort in the two exercise conditions.

An increasing number of studies have investigated the serum and plasma levels of BDNF during acute and chronic exercise in healthy subjects or in subjects with chronic diseases. Thus, these changes have been reported after endurance or high-intensity exercise (13–15). Therefore, increased blood BDNF may depend on the level of physical exertion as well as the status of training. To our knowledge, there is little consistent information to determine whether athletes' performance levels or specific sport modalities interfere with BDNF levels in different ways.

Three relevant aspects in our study that differ from other investigations should be considered. First, in the present work, a sport training session was performed under a real condition. This condition is more similar to a competition situation, a very common daily routine for high-performance athletes. Studies have investigated changes in neurotropic factors levels in athletes or trained subjects after a maximal physical effort (12,29). However, exercise execution in a laboratory environment cannot reflect the real condition during a specific type of training. The level, intensity, and training hours of elite athletes are somewhat different from healthy individuals, non-professional athletes, and those with some pathology. For instance, Judo is a dynamic, high-intensity sport that requires complex skills during training and competitions (30,31). Thus, the level of attention and the intensity of the exercise are fundamental in the sport’s performance. In our study, the athletes' weekly training load was approximately 25 h, which was rarely achieved by other studies. Here, we demonstrated that judo fights training (Randori) increased plasma levels of BDNF compared to basal levels. Our study is in line with Oztasyonar's (32) investigation, which showed higher serum BDNF levels after physical effort among middle- or long-distance running athletes, boxers, and taekwondo fighters. However, in his study, the training simulation was performed only for the fighter subjects. The boxers performed 3-min rounds for a total of 9 min training and the taekwondo subjects in 2x3 round training, while 1500-, 3000-, 5000-, and 10,000-m runners were submitted to 800 m running. Further investigations with highly competitive athletes are necessary to confirm our preliminary results.

Second, studies have recruited athletes from different modalities and different training levels (elite and non-elite) to evaluate the blood BDNF changes that can result in different outcomes. Few studies have involved well-trained athletes or healthy trained subjects, but not all were from the same athletic level (33,34). In Zoladz and collaborators' study (12), the BDNF values from both athletes at the national and international levels were analyzed together. Oztasyonar (32) reported that, among the runners and fighters from his sample, only boxers and taekwondo fighters were of national or international levels. Thus, these variables can distinctly alter circulating BDNF levels and consequently cannot provide an accurate correlation between BDNF levels and athletes' training status or their performance level as previously reported (12). To test this hypothesis, we chose to use a specific sport modality from world-ranked athletes. Our sample consisted of highly trained athletes from a long-term athletic training program and of international level. In line with our study, a recent investigation that analyzed the impact of neural plasticity on motor performance in elite swimmers ranked from 1st to 250th of the world found the strongest functional connectivity in athletes with the highest world rank (30).

With regard to basal concentration of BDNF in elite athletes, limited information has been reported, which does not allow us to draw a distinct conclusion. A higher basal plasma BDNF concentration was found in athletes (sprinters, jumpers, and runners) (12) and boxers and taekwondo fighters compared to untrained subjects (32). We have previously demonstrated that basal plasma BDNF increased in Brazilian sprint runners (100 m) of international level (Olympics and Outdoor World Championships) compared to domestic level or sedentary subjects (22). Unfortunately, we cannot make comparisons of the basal BDNF levels between elite athletes and sedentary subjects in the present study because we did not include an untrained control group in our sample.

The third important factor to be considered is the specificity of movement, that is, the impact of complex sport gestures in a specific sport modality and simple sport gestures on the BDNF blood levels in elite athletes. In our study, BDNF levels were higher after the judo training session than the ramp test and BDNF plasma variation after physical exercise increased significantly in both exercise conditions. Thus, delta BDNF from athletes increased approximately 260 and 485% 1 min post-exercise for the MIRT and Randori, respectively. This magnitude of increase seems to be higher than most studies, which have reported increases in BDNF on the order of 11 to 410% (15,26,35). However, several factors, such as intensity of physical exercise, small sample of subjects, and BDNF collection at different time-points after physical effort should be considered to draw accurate conclusions. Although in both types of exercise, all athletes reached at least 85% of HRmax, athletes performing MIRT achieved a higher heart rate than those submitted to the judo training session. A study comparing the efficacy of two protocols of high-intensity exercise showed that high-intensity interval training induced greater serum BDNF levels than continuous exercise (36). Considering judo as a dynamic modality comprising intermittent high intensity exercise that involves complex skills during training, it can be suggested that this type of sport modality brings on higher BDNF levels than cyclic sports or activities that require simple sport gestures. In accordance, Jacini and collaborators (27) reported higher gray matter volume in the brain of judo athletes compared to control subjects. These findings may reflect changes induced by motor tasks related to specificity of training that consequently alter the cerebral blood flow (37), neurotrophic factors, and brain metabolism (38).

We also analyzed whether the BDNF plasma concentration in elite athletes differed by gender. The basal BDNF was higher in females than in males for both exercise conditions. This difference was maintained post-Randori, but not post-MIRT. Nevertheless, the BDNF variation was similar in both exercise conditions. To our knowledge, no study has analyzed the impact of gender on BDNF levels in elite athletes. Some investigations with healthy subjects submitted to physical exercise have demonstrated contrasting results. For instance, sex differences in serum BDNF was found only during exercise (higher in men), but not at rest or post-exercise (39). Forti and collaborators (40) reported a significant increase in the BDNF levels in men but not in women after 12 weeks of a low-resistance training program. Interestingly, although not statistically significant, higher basal BDNF was found in female participants. In another study, the levels of BDNF were higher in men than women after 3 months of crossfit training (29). In Tang et al.'s study (34), no gender difference was found after physical effort; however, exercise condition (15 min moderate step exercise), blood taken after a long time post-effort, and the small sample size were interfering variables that may not provide reasonable results. Considering that BMI and individual fitness level were similar between genders, at least these variables did not interfere in our results. We should not discard differences in steroid levels. Unfortunately, we did not examine steroid concentrations in our sample. As previously pointed out (38), future investigations are needed to clarify the relationship between steroid levels and blood BDNF levels in elite athletes.

As a limitation of this investigation, we could not carry out a more complex study design (for example, a crossover study), because the participants were pre-selected for the national judo team and they needed to comply with the official training program and meeting dates.

In conclusion, although both types of physical effort induced a significant increase in plasma BDNF, higher BDNF levels were noted in athletes who were submitted to Randori than to MIRT. Training specificity and the type of sport gesture (simple or complex) influence the increase of blood BDNF levels. Further investigations using this neurotrophin as a brain biomarker should be explored focusing on performance enhancement.

## References

[1] van Praag H (2009). Exercise and the brain: something to chew on. Trends Neurosci.

[2] Cotman CW, Berchtold NC (2002). Exercise: a behavioral intervention to enhance brain health and plasticity. Trends Neurosci.

[3] Yacoubian TA, Lo DC (2000). Truncated and full-length TrkB receptors regulate distinct modes of dendritic growth. Nat Neurosci.

[B04] Alsina B, Vu T, Cohen-Cory S (2001). Visualizing synapse formation in arborizing optic axons in vivo: dynamics and modulation by BDNF. Nat Neurosci.

[B05] Kang H, Schuman EM (1995). Long-lasting neurotrophin-induced enhancement of synaptic transmission in the adult hippocampus. Science.

[B06] Seil FJ, Drake-Baumann R (2000). TrkB Receptor ligands promote activity-dependent inhibitory synaptogenesis. J Neurosci.

[B07] Friedman WJ (2000). Neurotrophins induce death of hippocampal neurons via the p75 receptor. J Neurosci.

[B08] Leibrock J, Lottspeich F, Hohn A, Hofer M, Hengerer B, Masiakowski P (1989). Molecular cloning and expression of brain-derived neurotrophic factor. Nature.

[9] Lim KC, Lim ST, Federoff HJ (2003). Neurotrophin secretory pathways and synaptic plasticity. Neurobiol Aging.

[10] Griesbach GS, Hovda DA, Molteni R, Wu A, Gomez-Pinilla F (2004). Voluntary exercise following traumatic brain injury: Brain-derived neurotrophic factor upregulation and recovery of function. Neuroscience.

[11] Molteni R, Wu A, Vaynman S, Ying Z (2004). Exercise reverses the harmful effects of consumption of a high-fat diet on synaptic and behavioral plasticity associated to the action of brain-derived neurotrophic factor. Neuroscience.

[12] Zoladz J, Fiore M, Majerczak J (2008). Endurance training increases plasma brain-derived neurotrophic factor concentration in young healthy men. J Physiol Pharmacol.

[13] Seifert T, Brassard P, Wissenberg M, Rasmussen P, Nordby P, Stallknecht B (2010). Endurance training enhances BDNF release from the human brain. Am J Physiol Regul Integr Comp Physiol.

[14] Ferris LT, Williams JS, Shen CL (2007). The effect of acute exercise on serum brain-derived neurotrophic factor levels and cognitive function. Med Sci Sport Exerc.

[15] Rojas S, Strüder HK, Vera B, Schmidt A, Bloch W, Hollmann W (2006). Acute BDNF and cortisol response to low intensity exercise and following ramp incremental exercise to exhaustion in humans. Brain Res.

[16] Yarrow JF, White LJ, McCoy SC, Borst SE (2010). Training augments resistance exercise induced elevation of circulating brain derived neurotrophic factor (BDNF). Neurosci Lett.

[17] Correia PR, Pansani A, Machado F, Andrade M, Silva AC, Scorza FA (2010). Acute strength exercise and the involvement of small or large muscle mass on plasma brain-derived neurotrophic factor levels. Clinics.

[18] Neeper SA, Gomez-Pinilla F, Choi J, Cotman C (1995). Exercise and brain neurotrophins. Nature.

[B19] Huang AM, Jen CJ, Chen HF, Yu L, Kuo YM, Chen HI (2006). Compulsive exercise acutely upregulates rat hippocampal brain-derived neurotrophic factor. J Neural Transm.

[20] Vaynman S, Gomez-Pinilla F (2005). License to run: exercise impacts functional plasticity in the intact and injured central nervous system by using neurotrophins. Neurorehabil Neural Repair.

[21] Vaynman S, Ying Z, Gomez-Pinilla F (2004). Hippocampal BDNF mediates the efficacy of exercise on synaptic plasticity and cognition. Eur J Neurosci.

[22] Correia PR, Scorza FA, Gomes S, Pansani A, Toscano-silva M, de Almeida AC (2011). Increased basal plasma brain-derived neurotrophic factor levels in sprint runners. Neurosci Bull.

[23] Ferris LT, Williams JS, Shen CL (2007). The effect of acute exercise on serum brain-derived neurotrophic factor levels and cognitive function. Med Sci Sport Exerc.

[B24] Winter B, Breitenstein C, Mooren FC, Voelker K, Fobker M, Lechtermann A (2007). High impact running improves learning. Neurobiol Learn Mem.

[25] Griffin EW, Mullally S, Foley C, Warmington SA, O'Mara SM, Kelly AM (2011). Aerobic exercise improves hippocampal function and increases BDNF in the serum of young adult males. Physiol Behav.

[26] Knaepen K, Goekint M, Heyman EM, Meeusen R (2010). Neuroplasticity - exercise-induced response of peripheral brain-derived neurotrophic factor: a systematic review of experimental studies in human subjects. Sports Med.

[27] Jacini WFS, Cannonieri GC, Fernandes PT, Bonilha L, Cendes F, Li LM (2009). Can exercise shape your brain? Cortical differences associated with judo practice. J Sci Med Sport.

[28] Wallwork SB, Bellan V, Catley MJ, Moseley GL (2016). Neural representations and the cortical body matrix: implications for sports medicine and future directions. Br J Sports Med.

[29] Murawska-Cialowicz E, Wojna J (2015). Crossfit training changes brain-derived neurotrophic factor and irisin levels at rest, after wingate and progressive tests, and improves aerobic capacity and body composition of young physically active men and women. J Physiol Pharmacol.

[30] Degoutte F, Jouanel P, Filaire E (2003). Energy demands during a judo match and recovery. Br J Sports Med.

[31] Franchini E, Del Vecchio FB, Matsushigue KA, Artioli GG (2011). Physiological profiles of elite judo athletes. Sport Med.

[32] Oztasyonar Y (2017). Interaction between different sports branches such as taekwondo, box, athletes and serum brain derived neurotrophic factor (BDNF) levels. J Sports Med Phys Fitness.

[33] Nofuji Y, Suwa M, Moriyama Y, Nakano H, Ichimiya A, Nishichi R (2008). Decreased serum brain-derived neurotrophic factor in trained men. Neurosci Lett.

[34] Kim Y (2016). The impact of exercise training on basal BDNF in athletic adolescents. J Phys Ther Sci.

[35] Tang SW, Chu E, Hui T, Helmeste D, Law C (2008). Influence of exercise on serum brain-derived neurotrophic factor concentrations in healthy human subjects. Neurosci Lett.

[36] Saucedo Marquez CM, Vanaudenaerde B, Troosters T, Wenderoth N (2015). High-intensity interval training evokes larger serum BDNF levels compared with intense continuous exercise. J Appl Physiol.

[37] Querido JS, Sheel AW (2007). Regulation of cerebral blood flow during exercise. Sport Med.

[38] Dishman RK, Berthoud HR, Booth FW, Cotman CW, Edgerton VR, Fleshner MR (2006). Neurobiology of Exercise. Obesity.

[39] Schmidt-kassow M, Schädle S, Otterbein S, Thiel C, Doehring A, Lötsch J (2012). Kinetics of serum brain-derived neurotrophic factor following low-intensity versus high-intensity exercise in men and women. Neuroreport.

[40] Forti LN, Van Roie E, Njemini R, Coudyzer W, Beyer I, Delecluse C (2015). Dose-and gender-specific effects of resistance training on circulating levels of brain derived neurotrophic factor (BDNF) in community-dwelling older adults. Exp Gerontol.

